# Clinical Outcome and Risk Factors of Red Blood Cell Transfusion in Patients Undergoing Elective Primary Meningioma Resection

**DOI:** 10.3390/cancers13143601

**Published:** 2021-07-18

**Authors:** Vanessa Neef, Sven König, Daniel Monden, Daniel Dubinski, Anika Benesch, Florian J. Raimann, Florian Piekarski, Michael W. Ronellenfitsch, Patrick N. Harter, Christian Senft, Patrick Meybohm, Elke Hattingen, Kai Zacharowski, Volker Seifert, Peter Baumgarten

**Affiliations:** 1 Department of Anaesthesiology, Intensive Care Medicine and Pain Therapy, University Hospital Frankfurt, Goethe University Frankfurt, 60590 Frankfurt, Germany; Vanessa.Neef@kgu.de (V.N.); Anika.Benesch@gmx.de (A.B.); Florian.Raimann@kgu.de (F.J.R.); Florian.Piekarski@kgu.de (F.P.); Meybohm_P@ukw.de (P.M.); Kai.Zacharowski@kgu.de (K.Z.); 2 Department of Neurosurgery, University Hospital, Goethe University Frankfurt, 60528 Frankfurt am Main, Germany; s8855616@stud.uni-frankfurt.de (S.K.); Daniel.Monden@med.uni-jena.de (D.M.); daniel.dubinski@med.uni-rostock.de (D.D.); christian.senft@med.uni-jena.de (C.S.); v.seifert@em.uni-frankfurt.de (V.S.); 3 Dr. Senckenberg Institute of Neurooncology, University Hospital Frankfurt, Goethe University Frankfurt, 60528 Frankfurt am Main, Germany; Michael.Ronellenfitsch@kgu.de; 4 Neurological Institute (Edinger Institute), University Hospital, Goethe University Frankfurt, 60528 Frankfurt am Main, Germany; patrick.harter@kgu.de; 5 Department of Neuroradiology, University Hospital, Goethe University Frankfurt, 60528 Frankfurt am Main, Germany; elke.hattingen@kgu.de

**Keywords:** anaemia, red blood cells, transfusion, meningioma

## Abstract

**Simple Summary:**

The transfusion of red blood cells (RBC) in patients undergoing major elective cranial surgery is associated with increased morbidity and mortality. This study sought to identify the clinical outcome of RBC transfusions in skull base and non-skull base meningioma patients including the identification of risk factors for RBC transfusion. Data underline that preoperative anaemia rate was significantly higher in transfused patients (17.7%) compared to patients without RBC transfusion (6.2%). We could further show that RBC transfusion was associated with increased postoperative complications and increased hospital length of stay. After multivariate analyses, risk factors for RBC transfusion were preoperative American Society of Anaesthesiologists (ASA) physical status score, tumor size, surgical time, and intraoperative blood loss. We concluded that blood loss due to large tumors or localization near large vessels are the main triggers for RBC transfusion in meningioma patients paired with a potential preselection that masks the effect of preoperative anaemia in multivariate analysis. So far, this has not been investigated in a large cohort (*n* = 423) of skull base and non-skull base meningioma patients.

**Abstract:**

Transfusion of red blood cells (RBC) in patients undergoing major elective cranial surgery is associated with increased morbidity, mortality and prolonged hospital length of stay (LOS). This retrospective single center study aims to identify the clinical outcome of RBC transfusions on skull base and non-skull base meningioma patients including the identification of risk factors for RBC transfusion. Between October 2009 and October 2016, 423 patients underwent primary meningioma resection. Of these, 68 (16.1%) received RBC transfusion and 355 (83.9%) did not receive RBC units. Preoperative anaemia rate was significantly higher in transfused patients (17.7%) compared to patients without RBC transfusion (6.2%; *p* = 0.0015). In transfused patients, postoperative complications as well as hospital LOS was significantly higher (*p* < 0.0001) compared to non-transfused patients. After multivariate analyses, risk factors for RBC transfusion were preoperative American Society of Anaesthesiologists (ASA) physical status score (*p* = 0.0247), tumor size (*p* = 0.0006), surgical time (*p* = 0.0018) and intraoperative blood loss (*p* < 0.0001). Kaplan-Meier curves revealed significant influence on overall survival by preoperative anaemia, RBC transfusion, smoking, cardiovascular disease, preoperative KPS ≤ 60% and age (elderly ≥ 75 years). We concluded that blood loss due to large tumors or localization near large vessels are the main triggers for RBC transfusion in meningioma patients paired with a potential preselection that masks the effect of preoperative anaemia in multivariate analysis. Further studies evaluating the impact of preoperative anaemia management for reduction of RBC transfusion are needed to improve the clinical outcome of meningioma patients.

## 1. Introduction

Preoperative anaemia is common in patients scheduled for major surgery, with a prevalence of up to 50% [1]. In patients undergoing a variety of non-neurosurgical procedures, recent studies revealed that preoperative anaemia is an independent risk factor for postoperative morbidity, mortality, prolonged hospital length of stay (LOS) and an increased risk for red blood cell (RBC) transfusions [2]. Given the sensitivity of the central nervous system to decreased oxygen delivery, especially neurosurgical patients have an increased anaemia vulnerability. This has been demonstrated in emergency situations like traumatic brain injury or subarachnoid haemorrhage [3,4]. Preoperative anaemia is frequent in patients undergoing major elective surgery with 28.7% [5]. A recent study by Bydon et al. revealed that of 8015 patients who underwent elective craniotomy for malignant tumor resection, 1710 patients (21.4%) were anaemic (anaemia defined by haemoglobin (Hb) value < 11 g/dL for women and <13 g/dL for men). In addition, anaemia was associated with increased 30-day morbidity and mortality in patients [6].

Overall, transfusion of RBC units remains the main treatment of anaemia in surgical patients. Complications associated with RBC transfusions itself are transfusion related lung injury, haemolytic reactions or transmission of infectious disease, among others [7]. Regarding patient’s clinical outcome RBC transfusions in elective cranial surgery are associated with prolonged hospital LOS, increased complication rate and 30-day mortality [8].

With a prevalence of almost one-third among brain tumors, meningiomas are one of the most frequent of all intracranial neoplasms most likely deriving from the meningothel of the arachnoid layer [9]. According to an analysis of patients who underwent craniotomy for resection of skull base meningiomas, 7 out 37 (18.7%) patients received RBC transfusions. Here, the transfused patients stayed longer in hospital compared to patients without RBC transfusion (19.9 vs. 4.9 days). In addition, the skull base was an independent risk factor for transfusion after considering meningioma size [10].

Until now, there are large numbers of studies focusing on the impact of anaemia and RBC transfusions in subarachnoid haemorrhage or traumatic brain injury [11,12,13,14]. In meningiomas so far, the impact of anaemia and RBC transfusions has only been investigated in a small patient population with skull base meningiomas [10].

This retrospective study aims to identify the clinical outcome of RBC transfusions in patients undergoing elective neurosurgical resection of skull base and non-skull base meningiomas including risk factors for RBC transfusion.

## 2. Materials and Methods

### 2.1. Patient Data

We retrospectively analyzed patients undergoing primary meningioma resection at the University Hospital Frankfurt from October 2009 to October 2016. All patients received standard perioperative care. Data were extracted from the electronic hospital information system.

Extracted data were preoperative patient specifics like age, sex, body mass index (BMI), American Society of Anaesthesiologists (ASA) physical status score, comorbidities and preoperative Karnofsky performance scale (KPS). For preoperative KPS we used a cutoff of ≤60% since that means independence of help for the daily life. Tumor related variables included prevalence of seizures, edema, embolization, tumor localization, histological brain invasion, histological subtype, Ki67-proliferation rate, mitosis count per 10 high-power fields (HPF), World Health Organization (WHO) grade and tumor size. WHO grade was histologically assessed according to the present WHO classification of central nervous system tumors [9]. Tumors were volumetriated on contrast enhanced T1 weighted magnetic resonance images (MRI) and edema was re-evaluated as suggested by Wirsching et al. on T2 weighted MRI images [15]. All WHO grades II and III meningiomas were histologically re-evaluated by at least two neuropathologists as published before in the same cohort [16].

Histological re-evaluation was performed with special attention on mitosis and histological brain invasion. Regarding surgical parameters, brain invasion defined as lack of arachnoid layer, and the extend of resection classified by the Simpson score were assessed. The Simpson score ranges from 1 to 5, and is defined as following: complete tumor removal and its attachments (1), complete tumor removal with denaturation of its attachments (2), complete intracranial tumor resection but leaving tumor in the cranial sinuus and/or without denaturation of attachments (3), incomplete tumor removal (4) and biopsy and/or decompression without tumor removal (5) [17]. Intraoperative parameters included intraoperative blood loss and surgical time. Postoperatively extracted data were hospital LOS, intensive care unit (ICU) LOS, and postoperative complications (acute renal failure, pneumonia, sepsis, pulmonary embolism, myocardial infarction, stroke, seizures, and re-craniotomy due to bleeding or swelling). Haematological and transfusion related parameters were Hb value at hospital admission and discharge as well as the perioperative transfusion rate of RBCs, platelets, fresh frozen plasma (FFP), fibrinogen and prothrombin complex concentrate (PCC). For follow up, the patients were seen in the outpatient clinic after three, nine, and twelve months. After this period, they were seen yearly or every two years depending on WHO grade and recurrent tumor growth.

### 2.2. Anaemia Classification

In the present study, anaemia was defined according to the WHO definition of anaemia. Here anaemia is defined as a Hb concentration of <12 g/dL in women and <13 g/dL in men [18].

### 2.3. Endpoints

Primary endpoints were the prevalence of preoperative anaemia and perioperative RBC transfusion rate. Secondary endpoints were hospital LOS and ICU LOS, postoperative complications, anaemia rate at hospital discharge and perioperative transfusion rate of other blood products (platelets, FFP, fibrinogen and PCC).

### 2.4. Statistical Analysis

Statistical analysis and figure editing were performed using JMP 14.0 software (SAS Institute, Cary, NC, USA), GraphPad Prism 6 (GraphPad Software Inc., La Jolla, CA, USA) and the open-source GIMP2 program. Evaluation of the immunohistochemical preparations was performed using a BX50 light microscope (Olympus, Tokyo, Japan). Tumor volumetriation was performed using the SmartBrush tool of the Brainlab Elements software (Brainlab AG, Munich, Germany).

Descriptive statistical methods mean (±SD) or median and interquartile range (IQR) (25–75%) were used to analyze data. Shapiro-Wilk test was used to assess normality of continuous variables. Normally distributed continuous variables (Hb value at hospital discharge) were compared with the two-sided T-test. Non-normally distributed continuous variables were compared with Mood’s median test. Categorical variables were compared with Pearson’s Chi Square test. For multivariate analysis, logistic regression was conducted. Survival analyses were performed using Kaplan-Meier analyses. To compare the survival curves, we used Wilcoxon and log-rank tests for censored data. A *p*-value < 0.05 was considered statistically significant.

## 3. Results

### 3.1. Patient Cohort

**Figure 1:**
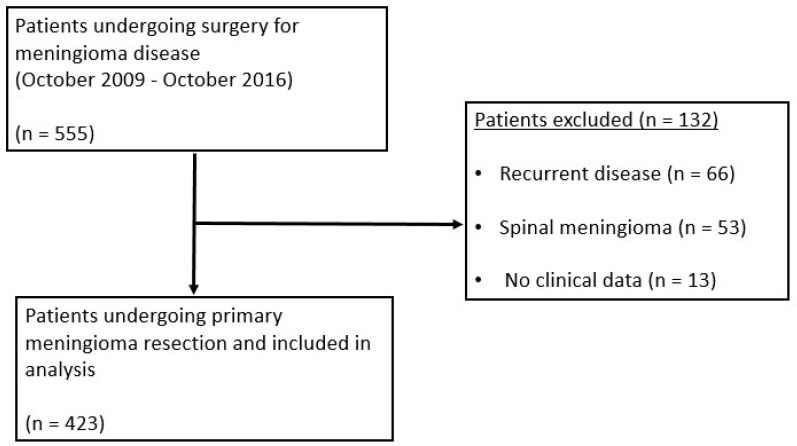
Flow chart of the selection process and overview of the study population.

Overall, 555 surgeries for meningioma disease were performed, of which 66 patients with recurrent tumors, 53 spinal cases and 13 patients with no preoperative Hb value were excluded from further evaluation. The remaining 423 patients were included in final analysis (Figure 1). The median follow-up period in this study cohort was 15 months (mean: 21.7 months, IQR: 12–35 months, range: 0–98 months).

### 3.2. Patient and Meningioma Characteristics Between the RBC Transfusion and Non-RBC Transfusion Group

Overall, 68 out of 423 (16.1%) patients received perioperative RBC transfusion and 355 out of 423 (83.9%) patients did not receive RBC transfusion. Median age in transfused patients was significantly higher (63 (51–71) years) compared to patients without RBC transfusion (56 (47–66) years; *p* = 0.0153). The rate of elderly patients (≥75 years) also differed significantly between the RBC transfusion group and non-RBC transfusion group (13.2% vs. 4.5%; *p* = 0.0068). The prevalence of comorbidities differed significantly between the two groups regarding cardiovascular disease (*p* = 0.0107) and diabetes (*p* = 0.0210). In addition, tumor size was significantly higher in the RBC transfusion group (64.9 (34.5–84.5) cm^3^) compared to the non-RBC transfusion group (19.2 (7.6–40.4) cm^3^; *p* < 0.0001) (Table 1).

### 3.3. Hb Values, Transfusion Rates and Postoperative Complications Between the RBC Transfusion and Non-RBC Transfusion Group

Overall, 34 out of 423 (8%) patients suffered from preoperative anaemia and 389 out of 423 patients (92%) did not have preoperative anaemia. Preoperative anaemia rate was significantly higher in the RBC transfusion group compared to the non-RBC transfusion group 17.7% (*n* = 12) vs. 6.2% (*n* = 22); *p* = 0.0015).

Intraoperatively, surgical time was significantly longer in patients with RBC transfusion (320 (259.2–377) min) compared to patients without RBC transfusion (217 (169–290) min; *p* < 0.0001). In addition, the amount of blood loss also differed significantly between both groups (1125 (525–1675) ml vs. 450 (300–1100) ml; *p* < 0.0001).

Regarding the transfusion rate of other blood products, transfusion rate of platelets (10.3% vs. 0.6%; *p* < 0.0001), FFP (14.7% vs. 0.3%; *p* < 0.0001), fibrinogen (27.9% vs. 1.4%; *p* < 0.0001) and PCC (14.7% vs. 2.5%; *p* = 0.0002) differed significantly between the RBC-transfusion and non-RBC transfusion group, respectively.

Hospital LOS was significantly longer in transfused patients (15.5 (10.3–23) days) compared to non-transfused patients (8 (7–11) days; *p* < 0.0001). In addition, postoperative complication rate was significantly higher in patients with RBC transfusion regarding acute renal failure (*p* = 0.0067), pneumonia (*p* < 0.0001), sepsis (*p* = 0.0067), pulmonary embolism (*p* = 0.0043), seizures (*p* = 0.0003) and re-craniotomy due to bleeding or swelling (*p* < 0.0001) compared to patients without RBC transfusion (Table 1).

### 3.4. Risk Factors for RBC Transfusions

For transfusion of RBC units in all patients (*n* = 423), the analyzed risk factors are summarized in Table 2. Of all analyzed factors, anaemia at hospital admission (*p* = 0.0015), age at surgery (*p* = 0.0153), elderly patients (≥ 75 years) (*p* = 0.0068), ASA score (*p* < 0.0001), cardiovascular disease (*p* = 0.0107), diabetes (*p* = 0.0210), KPS ≤ 60% (*p* = 0.0003), preoperative edema (*p* = 0.0079), tumor size (*p* < 0.0001), mitosis rate (*p* = 0.0040), surgical time (*p* < 0.0001) and intraoperative blood loss (*p* < 0.0001) were significantly associated with the risk of RBC transfusion. Multivariate analyses of all factors that showed significant influence in univariate analyses (*p* < 0.05) were performed, respectively. After logistic regression, ASA score (*p* = 0.0247), tumor size (*p* = 0.0006), surgical time (*p* = 0.0018) and intraoperative blood loss (*p* < 0.0001) remained as independent risk factors for perioperative RBC transfusion (Table 2).

**Figure 2:**
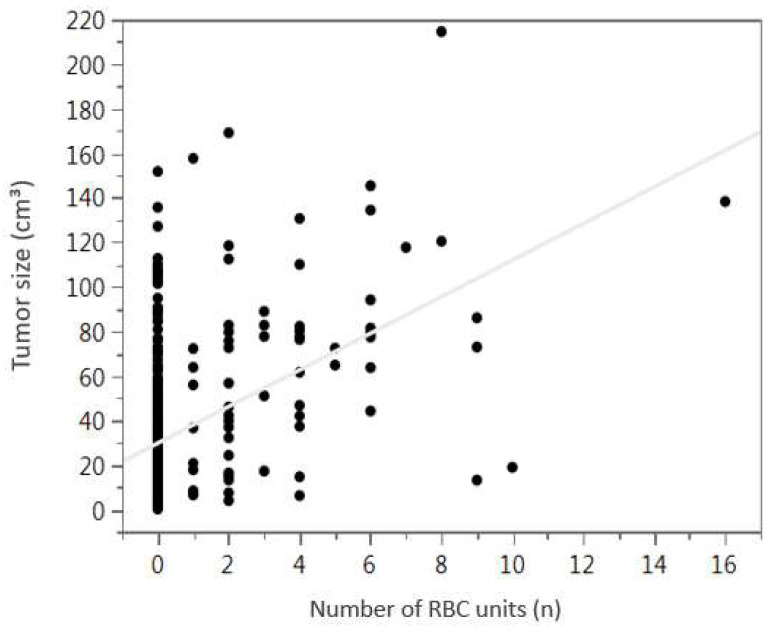
Simple linear regression illustrates the significant correlation of tumor size and number of transfused RBC units (R^2^ = 0.0183; *p* < 0.0001); RBC, red blood cell.

Furthermore, univariate linear regression analysis revealed a significant correlation between tumor size and number of transfused RBC units (R^2^ = 0.0183; *p* < 0.0001) (Figure 2).

### 3.5. Survival

**Figure 3:**
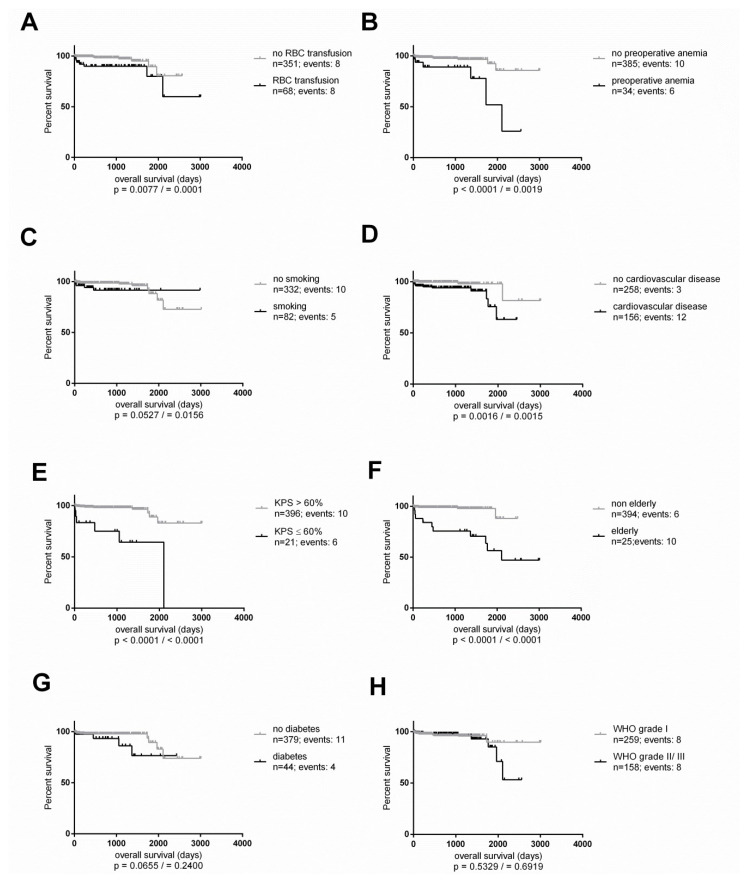
(**A**) Kaplan-Meier curve illustrates the overall survival in the cohort between patients with RBC transfusion and without RBC transfusion (**B**) Overall survival by preoperative anaemia and no preoperative anaemia (**C**) Overall survival by smoking and no smoking (**D**) Overall survival by cardiovascular disease and no cardiovascular disease (**E**) Overall survival by KPS > 60% and KPS ≤ 60% (**F**) Overall survival by elderly patients (≥75 years) and non-elderly patients (<75 years) (**G**) Overall survival by diabetes and no diabetes (**H**) Overall survival by WHO grade I and WHO grade II/III. *p*-values are obtained from Log-rank test/Wilcoxon Test.

Kaplan-Meier curves revealed significant influence on overall survival by RBC transfusion, preoperative anaemia, smoking, cardiovascular disease, preoperative KPS ≤ 60% and age (elderly patients ≥ 75 years). No influence was observed for diabetes and WHO grade (Figure 3).

## 4. Discussion

The present study examined the clinical outcome and risk factors of perioperative RBC transfusion in patients undergoing primary resection of skull base and non-skull base meningioma. Of all patients, 34 out of 423 (8%) were anaemic and 389 out of 423 (92.0%) did not suffer from preoperative anaemia. Overall, 68 out of 423 (16.1%) patients received RBC transfusion and 355 out of 423 (83.9%) patients did not receive RBC transfusion. Preoperative anaemia rate was significantly higher in the RBC transfusion group compared to the non-RBC transfusion group. Patients with RBC transfusion had increased postoperative complication rate and prolonged hospital LOS. Multivariate analyses revealed that ASA score, tumor size, surgical time and intraoperative blood loss are independent risk factors for RBC transfusion. In addition, Kaplan-Meier curves revealed significant influence on overall survival by preoperative anaemia, RBC transfusion, smoking, cardiovascular disease, preoperative KPS ≤ 60% and age (elderly ≥ 75 years).

### 4.1. Preoperative Anaemia and Clinical Outcome of RBC Transfusion

Recent findings in elective cranial neurosurgical patients have demonstrated, that preoperative anaemia is independently associated with an increased risk of 30-day postoperative mortality and morbidity compared to non-anaemic patients [6]. Furthermore, anaemia represents a major cause for RBC transfusion [19]. In our study, preoperative anaemia rate was low (8%) in patients undergoing primary craniotomy for meningioma disease. In comparison, anaemia rate in a study by Bydon et al. was higher with 21.4%. In this study, patients undergoing elective cranial neurosurgeries due to a variety of tumors were included [6]. The presented low preoperative anaemia rate in our study may also be explained by a potential preselection in neurosurgical patients since the natural course of meningioma disease allows elective planning of the operation. Patients with comorbidities associated with anaemia are often not suitable for cranial surgery and thus are put on a watch and wait strategy in a single case decision. It is noteworthy, that there are studies using different definitions of anaemia (e.g., by haematocrit) [20]. Our study defined anaemia according to the WHO definition by Hb value, therefore in these cases, any comparison has to be done carefully.

Overall, preoperative anaemia rate was significantly higher in the RBC transfusion group (17.7%) compared to the non-RBC transfusion group (6.2%; *p* = 0.0015). Similar results of anaemic patients being at greater risk for RBC transfusion were obtained in another study of patients undergoing elective cranial surgery. Here, RBC transfusion rates in severe-moderate and mild anaemia were significantly higher compared to non-anaemic patients, respectively (33.7% vs. 9.9% vs. 3.6%; *p* < 0.0001) [20]. In total, transfusion rate of all patients in our study was 16.1% (68 out of 423). These results are in accordance with a study by Langman et al., who evaluated 37 neurosurgical patients with skull base meningioma, whereas 7 out of 37 (18.9%) patients received RBC transfusion [10].

Our results also revealed that preoperative KPS ≤ 60 was more frequent in transfused patients (14.7%) compared to non-transfused patients (2.8%; *p* = 0.0003). Considering the fact that anaemia rate was higher in transfused patients, these results demonstrate that anaemia is accompanied with comorbidities and decreases the risk of functional decline in older adults. It is reported in literature that anaemia is associated with weakened muscle strength, mobility limitations and the ability to keep balance [21].

In our study patients with RBC transfusion had an increased risk of postoperative complications as well as prolonged hospital LOS. Similarly, Cohen et al. assessed the impact of RBC transfusion in cranial surgery through the National Surgical Quality Improvement Program database and demonstrated that patients who received RBC transfusion had an increased risk of postoperative complications, return to the operating room, prolonged hospital LOS, and higher mortality rates compared to non-transfused patients [8]. In general, patients who receive RBC transfusion are typically in a graver medical condition than those who do not [22].

Regarding overall survival, Kaplan-Meier curves revealed that preoperative anaemia and RBC transfusion had a significant influence on overall survival in meningioma patients. Since this is the first time investigating a large cohort of meningioma patients so far, comparison with other studies is not feasible.

### 4.2. Risk Factors of RBC Transfusion

In our study, univariate analyses revealed that preoperative anaemia, age (elderly ≥ 75 years), comorbidities (cardiovascular disease and diabetes), ASA score, preoperative edema, mitosis, tumor size, surgical time and intraoperative blood loss were a risk factors for RBC transfusion in meningioma patients. Multivariate analyses showed that ASA score, tumor size, surgical time and intraoperative blood loss remained as independent risk factors for RBC transfusion.

So far, there are only a few clinical studies investigating RBC transfusion in brain tumor surgery [10,23,24]. With a focus on independent risk factors for RBC transfusion in skull base meningiomas a study by Lagman et al [10]. showed that the skull base was an independent risk factor for RBC transfusion. In addition, a meningioma size greater than 5 cm was also a risk factor for transfusion. However, the authors state, that they were unable to determine whether tumor size was an independent risk factor, as sample size precluded multivariate analyses [10]. Our study includes 423 meningioma patients with skull base and non-skull base tumors and revealed tumor size as an independent risk factor for RBC transfusion. Especially large skull base meningiomas are often high vascularized and can enhance large vessels. Considering this fact, the intraoperative blood loss could be high since the vascular supply might be located within the tumor.

Our results demonstrate that there was a positive correlation between existing cardiovascular disease and the transfusion of RBC units. Especially in patients with underlying chronic cardiac condition, anaemia represents a risk factor for adverse cardiovascular events. Therefore, according to the German transfusion guidelines, RBC transfusion is recommended in patients with cardiovascular risk factors and limited compensatory capacity or in patients with clinical symptoms of anaemic hypoxia at Hb ≤ 8 g/dL. In patients without underlying cardiovascular disease or symptoms of impaired compensatory capacity, RBC transfusion is not recommended at Hb ≤ 8 g/dL [25]. This transfusion strategy in these patients may lead to higher RBC transfusion rates in patients with underlying cardiovascular disease in this study.

Simple linear regression also revealed a significant correlation of tumor volume and number of RBC units transfused which to our mind is comprehensible and highlights the reliability of our data.

In multivariate analyses preoperative anaemia did not remain an independent risk factor for RBC transfusion. In our study only 34 out of 423 (8%) patients were anaemic. Sample size did not preclude multivariate analyses. Here, blood loss due to large tumors or localization near large vessels is likely the main trigger for RBC transfusion in meningioma patients paired with a potential preselection that may mask the effect of preoperative anaemia in multivariate analysis.

Our data suggests that more patients were embolized in the transfusion group, however this did not reach statistical significance due to limited sample size of embolized patients. A study by Manaka et al. analyzed 69 patients with preoperative embolization for intracranial meningioma resection and revealed that preoperative embolization reduces intraoperative blood loss and surgical time by softening tumor consistency [26]. Considering our results of higher RBC transfusions in patients with larger tumors, greater amounts of intraoperative blood loss and longer surgical times, preoperative embolization of meningiomas may be helpful to reduce the need for allogeneic RBC units.

### 4.3. Strategies and Alternatives to RBC Transfusion

Our study revealed that RBC transfusion in meningioma patients is associated with an increased risk for postoperative complications and prolonged hospital LOS. In addition, RBC transfusion had significant influence on overall survival.

In order to minimize the risks associated with perioperative RBC transfusion, Patient Blood Management (PBM) has evolved within the last decade. PBM is an evidence-based, patient-centered and multidisciplinary approach in order to minimize the risks associated with anaemia in surgical patients [27]. The concept of PBM aims to reduce preoperative anaemia (pillar 1), minimize iatrogenic blood loss (pillar 2) and optimize patient specific tolerance of anaemia (pillar 3) in order to maintain the patient’s own blood volume and reduce allogeneic RBC transfusions [28].

As shown above, intraoperative blood loss represents an independent risk factor for perioperative RBC transfusion in multivariate analysis (*p* < 0.0001). Therefore, efforts should focus on reducing intraoperative iatrogenic blood loss and optimizing coagulopathy. Adequate surgical haemostasis, blood conservation techniques and coagulation management are seen as a precondition before RBC transfusion is considered. To optimize coagulopathy Meybohm et al. recommended the use of a coagulation algorithm, maintenance of basic conditions for haemostasis (body temperature > 36 °C, ionized calcium > 1.1 mmol/L, pH > 7.2) or point-of-care diagnostics.

Moreover, preoperative anaemia was associated with an increased number of RBC transfusions in univariate analysis (*p* = 0.0015). Consequently, preoperative anaemia management should be considered in meningioma patients and the cause of anaemia should be addressed accordingly. Depending on the cause of preoperative anaemia, the most common ways to treat preoperative anaemia are the use of iron supplementation and erythropoiesis stimulating agents [29]. In case of preoperative iron deficiency anaemia (IDA), recent studies proved that the administration of intravenous iron reduces the need for RBC transfusion and is associated with a shorter hospital LOS [30,31]. Triphaus et al. evaluated the effect of iron supplementation in patients with IDA undergoing major elective surgery. All iron-supplemented IDA patients required less RBC units during the postoperative period (31.5%) compared to anaemic patients without iron treatment (42.5%). In addition, hospital LOS was significantly reduced by 2.8 days in iron-supplemented IDA patients (13.9 (±0.8) days) compared to anaemic patients without iron treatment (16.7 (±0.7); *p* < 0.01) [19].

Especially in neurosurgical patients, preoperative strategies for identification and treatment of preoperative anaemia as well as intraoperative strategies like correction of coagulopathy or use of anti-fibrinolytic drugs are highly important [32]. Studies revealed that anaemia contributes to reduced cerebral oxygen delivery with secondary hypoxic insult to the injured brain. In healthy volunteers, anaemia-induced cognitive dysfunction is evident at Hb value between 5.0 and 6.0 g/dl [33].

Finally, RBC transfusions have shown to significantly increase postoperative morbidity and mortality, therefore putting an enormous pressure on health care facilities [34]. With focus on preoperative anaemia correction, an assessment of cost benefits by intravenous iron supplementation prior to elective abdominal surgery has demonstrated hospital cost savings of €786 per case based on the reduced number of transfused RBC units and shorter hospital LOS [35].

### 4.4. Limitations

Our study is limited by the retrospective, single center design. In addition, the cause of preoperative anaemia cannot be discerned with the database, however ID may be a potential and common cause of anaemia. Furthermore, the anaemia-rate is quite low and introduces a selection bias compared to population-based studies. Furthermore, the effectiveness of presurgical embolization in reducing RBC transfusion requirements during elective meningioma resection should be evaluated in future studies.

## 5. Conclusions

In conclusion, the number of anaemic patients was significantly higher in the transfused patient group. We identified several risk factors for RBC transfusion, which include ASA score, tumor size, surgical time and intraoperative blood loss. Regarding clinical outcome, transfused patients had longer hospital LOS and higher postoperative complication rates. In the past, studies on patients undergoing major surgical procedures have demonstrated that anaemia and RBC transfusions are associated with increased postoperative complications, morbidity and mortality. Especially in a neurosurgical patient population, preoperative anaemia identification and treatment is crucial. Future prospective studies need to evaluate the potential clinical benefit of preoperative anaemia management as part of a holistic PBM program in meningioma patients if anaemia or large tumors are present. Preoperatively, consideration should be given to the correction of underlying coagulopathy and anaemia, by the use of iron replacement or erythropoietin. Intraoperatively, the use of anti-fibrinolytic drugs or continuous Hb monitoring should also be considered in future trials. Last, the association of different anaemia grades on clinical outcome and transfusion requirements should be considered in future analyses.

## Figures and Tables

**Table 1:** Demographic and meningioma data, postoperative complications, and transfusion rates between the RBC transfusion and non-RBC transfusion group.

Characteristic	RBC-Transfusion *n* (%) 68 (16.1%)	Non-RBC Transfusion *n* (%) 355 (83.9%)	*p*-Value
Male/Female	25 (36.8%)/43 (63.2%)	112 (31.5%)/243 (68.5%)	=0.4039
Age (years) *	63 (51–71)	56 (47–66)	=0.0153
Elderly (≥75 years)	9 (13.2%)	16 (4.5%)	=0.0068
BMI (kg/m^2^) *	26.3 (22.8–38.9)	26.3 (23.1–33.5)	=0.9825
ASA score **	1 (1.8%)/24 (42.1%)/30 (52.6%)/2 (3.5%)	45 (15.0%)/181 (60.3%)/73 (24.3%)/1 (0.3%)	<0.0001
Comorbidities			
Smoking	14 (20.6%)	69 (19.4%)	=0.8264
Cardiovascular disease	35 (51.5%)	124 (34.9%)	=0.0107
Pulmonary disease	8 (11.8%)	23 (6.6%)	=0.1583
Diabetes	13 (19.1%)	32 (9.1%)	=0.0210
Endocrine disorders	28 (41.2%)	105 (29.6%)	=0.0676
Chronic kidney injury	21 (30.9%)	94 (26.6%)	=0.4273
Coagulopathy	2 (2.9%)	2 (0.6%)	=0.1167
Preoperative seizure **	16 (25%)	80 (23.5%)	=0.8008
Meningioma			
Non-Skull-Base/Skull-Base	40 (58.8%)/28 (41.2%)	231 (65.1%)/124 (34.9%)	=0.3290
Tumor size (cm^3^) *	64.9 (34.5–84.5)	19.2 (7.6–40.4)	<0.0001
Near large vessel	28 (40.2%)	107 (30.1%)	=0.0787
Brain invasion **	16 (26.2%)	63 (21.1%)	=0.3900
WHO grade I vs. II/III	35 (51.5%)/33 (48.5%)	227 (63.9%)/128 (36.4%)	=0.0615
Simpson grade <III/≥III **	18 (58%)/13 (41.9%)	81 (73.6%)/29 (26.4%)	=0.1013
Ki67 (%) *	3 (3–5)	3 (3–5)	=0.3223
Mitosis <4/10HPF/4–20/10HPF/ >20/10HPF **	39 (69.6%)/24 (42.9%)/3 (5.4%)	268 (78.4%)/70 (20.5%)/4 (1.2%)	=0.0040
Preoperative edema Non/≤tumor volume/ >tumor volume **	14 (21.5%)/29 (44.6%)/22 (33.8%)	136 (41.3%)/107 (32.5%)/86 (26.1%)	=0.0079
Preoperative KPS ≤ 60%	10 (14.7%)	10 (2.8%)	=0.0003
Surgical Management			
Embolization	1 (1.5%)	0 (0%)	=0.0555
Surgical time (min) *	320 (259.2–377)	217 (169–290)	<0.0001
Intraoperative blood loss (mL) *	1125 (525–1675)	450 (300–1100)	<0.0001
Postoperative Complications			
Acute renal failure	2 (2.9%)	0 (0%)	=0.0067
Pneumonia	13 (19.1%)	5 (1.4%)	<0.0001
Sepsis	2 (2.9%)	0 (0%)	=0.0067
Pulmonary embolism	10 (14.7%)	16 (4.5%)	=0.0043
Seizure **	21 (36.8%)	50 (15.2%)	=0.0003
Re-craniotomy **	22 (41.5%)	36 (11.4%)	<0.0001
Hospital LOS (days) *	15.5 (10.3–23)	8 (7–11)	<0.0001
ICU LOS (days) *	6 (2–15)	1 (1–1)	<0.0001
Hb values and Transfusion rates			
Hb value (admission) (g/dL) *	13.8 (12.8–14.7)	14.1 (13.3–14.8)	=0.1072
Anaemia rate at admission	12 (17.7%)	22 (6.2%)	=0.0015
Hb value (discharge) (g/dL) *	9.8 (8.7–10.5)	11 (10–12)	<0.0001
Anaemia rate at discharge	66 (97.0%)	290 (81.6%)	=0.0002
Platelet transfusion rate	7 (10.3%)	2 (0.6%)	<0.0001
FFP transfusion rate	10 (14.7%)	1 (0.3%)	<0.0001
Fibrinogen transfusion rate	19 (27.9%)	5 (1.4%)	<0.0001
PCC transfusion rate	10 (14.7%)	9 (2.5%)	=0.0002

* Results are expressed as median (IQR). BMI, Body Mass Index; ASA, American Society of Anaesthesiologists; WHO, World Health Organization; KPS, Karnofsky performance scale; HPF, High power fields; Hb, Haemoglobin; RBC, Red blood cell; FFP, Fresh frozen plasma; ICU, Intensive care unit; LOS, Length of stay; PCC, Prothrombin complex concentrate. Note: Bold *p*-value = statistically significant. ** Data not from all patients available.

**Table 2:** Risk factors for RBC transfusion.

Risk Factor	Univariate Analysis	Multivariate Analysis			
	*p*-Value	*p*-Value	OR	RR	95%CI
Anaemia at admission	=0.0015	=0.0900	1.02	2.77	−0.12–1.46
Age	=0.0153	=0.5567	0.36	0.67	−0.07–0.03
Elderly (≥75 years)	=0.0068	=0.0905	0.46	0.90	−0.47–1.31
ASA score	<0.0001	=0.0247	1.48	8.74	
ASA score 2–1					−4.84–0.62
ASA score 3–2					−2.02–0.08
Cardiovascular disease	=0.0107	=0.0700	1.21	3.47	−0.03–1.07
Diabetes	=0.0210	=0.4369	0.47	0.92	−1.01–0.31
Preoperative KPS ≤ 60%	=0.0003	=0.2309	0.52	1.08	−0.39–1.24
Preoperative edema	=0.0079	=0.7438	0.16	0.76	
≤tumor volume-Non					−1.41–1.02
>tumor volume ≤tumor volume					−0.66–1.78
Tumor size Mitosis	<0.001 =0.0040	=0.0006 =0.3686	3.03 0.24	10.10 1.10	−0.04–0.01
>4–20/10HPF <4/10HPF					−1.56–0.73
>20/10HPF 4–20/10HPF					−3.64–2.67
Surgical time	<0.0001	=0.0018	2.11	7.10	−0.01–0.00
Intraoperative blood loss	<0.0001	<0.0001	4.38	16.80	−0.01–0.00

Note: *p*-value = statistically significant in univariate analysis, underlined bold *p*-value = statistically significant in multivariate analysis; ASA, American Society of Anaesthesiologists; KPS, Karnofsky performance scale; HPF, High power fields; OR, Odds Ratio; RR, Risk Ratio; CI, Confidence interval.

## Data Availability

The data presented in this study are available on request from the corresponding author. The data are not publicly available due to ethical restrictions.
